# Baseline Cytomegalovirus Immunoglobulin G Levels Predict Reactivation in Patients With Multiple Myeloma Receiving Elranatamab

**DOI:** 10.1002/jha2.70189

**Published:** 2025-11-28

**Authors:** Ukyo Kondo, Taku Kikuchi, Shotaro Sugita, Miyu Watanabe, Chiaki Matsumoto, Moe Nomura‐Yogo, Kodai Kunisada, Kota Sato, Tomomi Takei, Mizuki Ogura, Yu Abe, Osamu Hosoya, Tadao Ishida, Nobuhiro Tsukada

**Affiliations:** ^1^ Department of Hematology Japanese Red Cross Medical Center Shibuya Tokyo Japan; ^2^ Department of Pharmacy Japanese Red Cross Medical Center Shibuya Tokyo Japan

**Keywords:** CMV IgG, cytomegalovirus, elranatamab, multiple myeloma

## Abstract

**Background:**

The cumulative incidence and risk of cytomegalovirus (CMV) reactivation in patients with relapsed/refractory multiple myeloma (RRMM) receiving elranatamab remain unclear.

**Methods:**

We retrospectively analyzed RRMM patients treated with elranatamab to assess cumulative incidence and risk factors for CMV reactivation.

**Results:**

Among 32 patients, the 6‐month cumulative incidence of CMV reactivation was 37.3%. High CMV immunoglobulin G (IgG) titers were significantly associated with CMV reactivation (57.9% vs. 9.1%, *p* = 0.012), while prophylactic immunoglobulin reduced CMV reactivation risk within high CMV IgG group.

**Conclusion:**

Baseline CMV IgG titers may help guide risk‐adapted monitoring and prevention strategies during elranatamab therapy.

1

Dear editor;

The bispecific antibody elranatamab is effective against triple‐class‐exposed relapsed or refractory multiple myeloma (RRMM). This therapy targets CD3 and B‐cell maturation antigen, and is associated with adverse events such as cytokine release syndrome (CRS), immune effector cell‐associated neurotoxicity syndrome, cytopenia, and hypogammaglobulinemia, which may affect treatment outcomes [1, 2]. Although cytomegalovirus (CMV) reactivation is increasingly recognized as a significant infectious complication, its cumulative incidence and associated risk factors remain unclear [3, 4]. This study aimed to evaluate the cumulative incidence of CMV reactivation during elranatamab therapy and to assess whether baseline CMV immunoglobulin G (IgG) titers could predict the risk of reactivation.

We retrospectively analyzed the electronic medical records of patients with RRMM who received initial elranatamab therapy at the Japanese Red Cross Medical Center (JRCMC).

CMV seropositivity was defined as a CMV IgG level of ≥ 6.0 AU/mL, measured via chemiluminescent immunoassay before elranatamab administration. CMV deoxyribonucleic acid (DNA) was measured in plasma using a commercial real‐time polymerase chain reaction (PCR) assay (detection limit: 35 IU/mL). CMV reactivation was defined as a CMV DNA level exceeding 500 copies/mL. In cases where CMV DNA was not measured, CMV reactivation was defined by the presence of ≥ 7 pp65 antigen‐positive cells per 50,000 cells. While weekly CMV PCR testing was preferred following elranatamab administration, the testing frequency was at the discretion of the attending physician. Fisher's exact test was used for comparisons between categorical variables, while Student's *t*‐test or the Mann–Whitney *U* test was used for continuous variables. The cumulative incidence of CMV reactivation was estimated using Gray's test, considering relapse or death before CMV reactivation as competing risks. Statistical significance was defined as *p* < 0.05. All statistical analyses were performed using EZR software (https://www.jichi.ac.jp/saitama‐sct/SaitamaHP.files/statmedEN.html) [5].

A total of 32 patients received elranatamab therapy between June 2024 and July 2025. None of the patients met the protocol‐defined criteria for CMV reactivation at the initiation of elranatamab. CMV IgG titers were measured in 27 patients, whereas the remaining 5 had unknown CMV IgG status. Patient characteristics are summarized in Table 1. The median age was 65.5 (range: 42–80) years, and 15 (46.9%) patients were female. The median CMV IgG titer was 166.10 AU/mL (range: 0–250). The cumulative incidence of CMV reactivation was 26.5% (95% confidence interval [CI], 12.5%–42.7%) at 3 months and 37.3% (95% CI, 20.4%–54.3%) at 6 months (Figure 1A). Preemptive therapy with valganciclovir was initiated when CMV PCR levels exceeded 500 copies/mL, resulting in a subsequent decline in viral load. Among patients who remained on elranatamab after discontinuing antiviral therapy, 9 exhibited no CMV reactivation. In contrast, 4 patients developed reactivation that required retreatment. Furthermore, 1 patient developed CMV retinitis. Receiver operating characteristic curve analysis identified 88.7 AU/mL as the optimal threshold of CMV IgG titer for predicting CMV reactivation. Patients were classified into high (≥ 88.7 AU/mL) and low (< 88.7 AU/mL) CMV IgG groups. No significant differences between the groups in the frequency or duration of CMV monitoring or in baseline characteristics were observed. The cumulative incidence of CMV reactivation at both 3 and 6 months was 9.1% (95% CI, 0.5%–33.3%) in the low CMV IgG group. In contrast, the incidence was higher in the high CMV IgG group, reaching 42.2% (95% CI, 17.3%–65.4%) at 3 months and 57.9% (95% CI, 28.3%–78.9%) at 6 months (*p* = 0.012) (Figure 1B). In the overall cohort, none of the following factors were significantly associated with the cumulative incidence of CMV reactivation: prophylactic immunoglobulin administration (*p* = 0.68), prior chimeric antigen receptor (CAR)‐T cell therapy (*p* = 0.53), and use of tocilizumab (*p* = 0.91) or corticosteroids for CRS (*p* = 0.18). However, among patients with the high CMV IgG group, those who received prophylactic immunoglobulin after elranatamab therapy had a significantly lower 3‐month CMV reactivation rate than those who did not (27.3% vs. 100%, *p* < 0.01) (Figure 1C). Given the small subgroup size, this finding is exploratory.

**TABLE 1:** Patient characteristics.

Variable	Overall *N *= 32	Low CMV IgG *N *= 11	High CMV IgG *N *= 16	*p* value
Age (years), median (range) at the time of elranatamab	65.5 (42–80)	60 (42–73)	69 (53–80)	0.071
Sex, *n* (%)				
Female	15 (46.9%)	6 (54.6%)	6 (62.5%)	0.45
MM subtype, *n* (%)				0.37
IgG	12 (43.8%)	5 (45.5%)	9 (56.3%)	
IgA	6 (18.8%)	3 (27.3%)	1 (6.3%)	
Bence‐Jones	14 (37.5%)	3 (27.3%)	6 (37.5%)	
CMV IgG titer (AU/mL) , median (range)	166.10 (0–250)	23.8 (0–88.7)	214.6 (98.3–250)	< 0.01
Prior autologous stem cell transplantation, *n* (%)	18 (56.3%)	6 (54.6%)	9 (56.3%)	1
Prior allogeneic stem cell transplantation, *n* (%)	2 (6.3%)	0 (0%)	2 (12.5%)	0.50
Prior chimeric antigen receptor T‐cell, *n* (%)	5 (15.6%)	2 (18.2%)	2 (12.5%)	1
CRS	18 (56.3%)	6 (54.6%)	10 (62.5%)	0.71
Tocilizumab use for CRS	15 (46.9%)	6 (54.6%)	7 (43.8%)	0.70
Corticosteroid use for CRS	2 (6.3%)	1 (9.1%)	0 (100%)	0.41
ICANS	1 (3.2%)	1 (9.1%)	0 (0%)	0.42
Prophylactic Ig	15 (46.9%)	4 (36.4%)	11 (68.8%)	0.13
High‐risk cytogenetic abnormality until elranatamab therapy	22 (68.8%)	9 (81.8%)	9 (56.3%)	0.18
t(4;14)	8 (25.0%)	5 (45.5%)	1 (6.3%)	0.027
t(14;16)	2 (6.3%)	1 (9.1%)	0 (0%)	0.41
del(17p)	8 (25.0%)	4 (36.4%)	4 (25%)	0.68
1q abnormality	20 (62.5%)	8 (72.7%)	8 (50%)	0.43
Prior treatment lines, median (range)	5 (2–9)	5 (2–7)	5 (3–9)	0.34
Penta‐class refractory^a^	15 (46.9%)	4 (36.4%)	6 (46.2%)	0.70
R2‐ISS stage at elranatamab, *n* (%)				0.84
Stage I	3 (9.4%)	1 (9.1%)	2 (12.5%)	
Stage II	4 (12.5%)	1 (9.1%)	3 (18.8%)	
Stage III	16 (50.0%)	4 (36.4%)	7 (43.8%)	
Stage IV	9 (28.1%)	5 (45.5%)	4 (25%)	
Lymphocytes at elranatamab (/µL), median (range)	690 (81–2480)	553.5 (81–1630)	900 (210–2480)	0.057

Abbreviations: CAR‐T, chimeric antigen receptor T‐cell; CMV, cytomegalovirus; CRS, cytokine release syndrome;

ICANS, immune effector cell‐associated neurotoxicity syndrome; Ig, immunoglobulin; IgA, immunoglobulin A; IgD, immunoglobulin D; IgG, immunoglobulin G; MM, multiple myeloma; R2‐ISS, second revision of the international staging system.

^a^ Penta‐class refractory is defined as refractory to two proteasome inhibitors, two immunomodulatory drugs, and an anti‐CD38 antibody.

**FIGURE 1:**
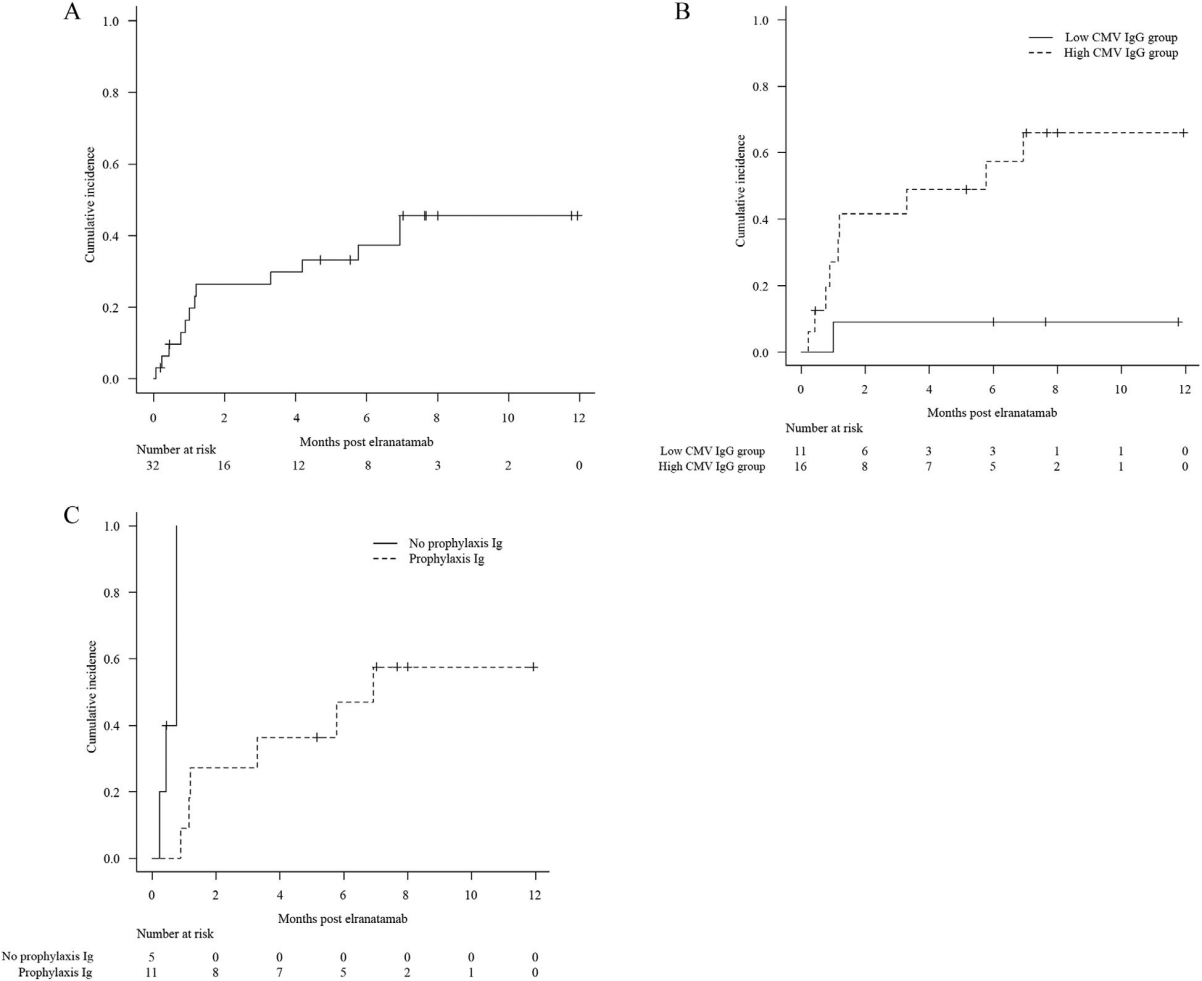
Cumulative incidence of cytomegalovirus reactivation (A) in all patients, (B) stratified by high versus low cytomegalovirus IgG titers, and (C) in patients with high cytomegalovirus IgG titers, stratified by prophylactic immunoglobulin use.

Among 13 patients with CMV reactivation, 4 experienced recurrent reactivation after initial viral clearance with preemptive antiviral therapy and required reinitiation of treatment. Preemptive therapy was defined as initiating antiviral drug upon detection of CMV DNA and discontinued once CMV DNA became undetectable. These findings suggest that continued CMV PCR monitoring may be warranted even after discontinuation of antiviral therapy. In CAR‐T therapy, treatment is completed with a single infusion, and CMV reactivation typically plateaus [6, 7]. In contrast, elranatamab is administered continuously, potentially resulting in prolonged immunosuppression and an increase in CMV reactivation risk over time. Furthermore, this study demonstrated that patients with high baseline CMV IgG titers had a significantly increased risk of CMV reactivation. This observation is consistent with previous findings in allogeneic hematopoietic stem cell transplantation and CAR‐T therapy, suggesting that high CMV IgG titers may be a common risk factor for reactivation across treatment settings [8, 9]. In the high CMV IgG group, prophylactic immunoglobulin use was associated with a significantly lower rate of CMV reactivation. Given the limited sample size, further studies are required to validate these findings.

This study has limitations as a single‐center, retrospective study with a limited sample size. Nevertheless, baseline CMV IgG titers may help identify patients at high risk of CMV reactivation before elranatamab initiation. This stratification could support risk‐adapted CMV PCR monitoring and consideration of prophylactic immunoglobulin administration to reduce the incidence of CMV disease. Although prophylactic use of letermovir, a selective inhibitor of the CMV DNA terminase complex, has not yet been established in patients treated with bispecific antibodies, its demonstrated efficacy in allogeneic stem cell transplantation recipients suggests a potential role in elranatamab therapy that warrants further study [10]. These findings may contribute to the development of improved strategies for CMV reactivation, including risk‐based monitoring and prevention.

## Author Contributions

Ukyo Kondo and Taku Kikuchi treated the patients and wrote the manuscript. Nobuhiro Tsukada treated the patients and provided important opinions regarding the study. Shotaro Sugita, Miyu Watanabe, Kodai Kunisada, Moe Nomura‐Yogo, Kota Sato, Tomomi Takei, Mizuki Ogura, Yu Abe, and Tadao Ishida treated the patients. Chiaki Matsumoto and Osamu Hosoya were responsible for data collection. All authors critically reviewed and approved the final version of the manuscript.

## Funding

The authors have nothing to report.

## Ethics Statement

This study was approved by the Institutional Review Board of the JRCMC (Tokyo, Japan, approval number: 1736) and conducted under an abbreviated informed consent procedure and an opt‐out consent principle. No participants were excluded from the study. This study was conducted in accordance with the principles of the Declaration of Helsinki.

## Conflicts of Interest

Taku Kikuchi received personal fees from Janssen Pharmaceuticals (Titusville, NJ, USA), Takeda (Osaka, Japan), Bristol‐Myers Squibb (New York, NY, USA), Pfizer (New York, NY, USA), and Sanofi (Paris, France). Nobuhiro Tsukada receives personal fees from Janssen and Sanofi. Tadao Ishida received honoraria from Ono Pharmaceutical Co., Ltd. (Osaka, Japan), Takeda, Celgene/Bristol‐Myers Squibb, and Janssen. The other authors declare no conflicts of interest.

## Data Availability

The datasets generated and analyzed in the present study are available from the corresponding author upon reasonable request.
